# Unveiling Psychiatric Complexities in 48,XXYY Syndrome: A Case Study

**DOI:** 10.7759/cureus.90743

**Published:** 2025-08-22

**Authors:** Tyler Francisco, Aura C Spar, Katherine M Napalinga

**Affiliations:** 1 Psychiatry, Drexel University College of Medicine, West Reading, USA; 2 Psychiatry, Tower Health Medical Group, West Reading, USA

**Keywords:** anxiety, autism spectrum disorder, depression, klinefelter syndrome, xxyy syndrome

## Abstract

The 48,XXYY syndrome sex chromosome variation is a genetic condition defined by the presence of an extra X and Y chromosome, confirmed with karyotype testing. Despite their similarities, 48,XXYY syndrome is distinct from Klinefelter syndrome on account of several factors, including psychiatric ones. This case report focuses on an individual with a history of 48,XXYY syndrome, autism spectrum disorder (ASD), and attention-deficit/hyperactivity disorder (ADHD) who had multiple psychiatric presentations for worsening depression and suicidal ideation. This case explores some of the comorbid psychiatric conditions considered in this patient’s presentations and reviews the existing literature to contemplate the differential diagnosis. The individual in this case exhibited behaviors linked to ASD or underlying personality disorder traits that initially complicated treatment, but with increasing familiarity and collateral history, appropriate recommendations were made. 48,XXYY syndrome warrants further interest, as it has had limited coverage in the literature. It would be valuable to examine how this patient's psychiatric profile compares to those of patients with other extra X or Y chromosome syndromes.

## Introduction

The 48,XXYY (or XXYY) syndrome is a type of sex chromosome aneuploidy (SCA). Initially referred to as the "double male," this condition is defined by the presence of two additional sex chromosomes in males, leading to a 48,XXYY karyotype [1]. Although it was once thought to be a variant of Klinefelter syndrome (47,XXY), 48,XXYY syndrome is now acknowledged as a distinct clinical condition due to its unique and more complex characteristics [2].

Klinefelter syndrome, defined by a 47,XXY karyotype, is the most common sex chromosome aneuploidy and is often associated with advanced maternal age [3]. It typically leads to primary hypogonadism, with hormonal imbalances including decreased testosterone and increased levels of follicle-stimulating hormone (FSH) and luteinizing hormone (LH) [4]. Physical manifestations include tall stature, gynecomastia, and testicular atrophy, along with increased risks of osteoporosis and certain cancers [3].

While there is significant overlap of physical and psychiatric manifestations, 48,XXYY syndrome notably presents with more severe developmental, intellectual, and behavioral challenges. Individuals with this syndrome often exhibit lower verbal IQ compared to performance IQ, significant developmental delays, and a higher prevalence of learning disabilities [5]. Behavioral issues such as attention-deficit/hyperactivity disorder (ADHD) and increased aggression are more common in 48,XXYY syndrome, with over 70% of individuals affected by ADHD [6]. Additionally, there is an increased prevalence of autism traits in individuals with 48,XXYY syndrome, with research indicating higher levels of autism traits across all dimensions compared to control groups [7]. Language deficits and impairments in social cognitive processing are more pronounced in 48,XXYY syndrome, contributing to significant social dysfunction [7].

This case report focuses on an individual with 48,XXYY syndrome and co-occurring autism spectrum disorder (ASD), who presented with multiple psychiatric symptoms, including worsening depression and suicidal ideation (SI). His clinical presentation underscores the importance of recognizing 48,XXYY syndrome as a distinct condition, requiring tailored approaches to management and support.

## Case presentation

Initial presentation

The patient highlighted in this case is a 21-year-old male subject with 48,XXYY syndrome (diagnosed at 13 months old) who initially presented to the crisis center with reports of increased SI and self-injurious behavior via superficial cuts on the wrist. Patient reported worse SI for the past 48 hours. He also reported worsening auditory hallucinations of voices talking to each other, discussing suicide plans, and intermittently commanding him to act on them. He states the auditory hallucinations started three years prior, but he did not further elaborate on frequency or other qualities. Plans for suicide included driving his car off the road, overdosing on his medications, aggravating police officers, and being shot, inducing anaphylaxis by eating peanuts (as he is known to be highly allergic), or using a razor blade to cut his wrists and throat. He reported a history of one previous suicide attempt via cutting his wrists to bleed out eight months ago. He also stated he had worsening impulsive behavior, such as drinking alcohol and then driving afterwards. He did not specify the frequency of impulsive behavior, but reported drinking one mixed alcoholic beverage once every two to three nights, with one instance of drinking four mixed alcoholic beverages in one night in the past year. He had been previously prescribed venlafaxine ER (Effexor XR) 150 mg daily with breakfast and prazosin 2 mg nightly for depression and posttraumatic stress disorder (PTSD), which he reports taking consistently for at least the past six months. He denied a history of abuse, but reported unspecified trauma from a previous inpatient hospitalization in the past year, where he experienced frequent nightmares. The patient had also been utilizing testosterone 20.25 mg/1.25 g (1.62%) transdermal gel for testosterone replacement for Klinefelter syndrome with no recent changes. Mental status exam demonstrated no appreciated abnormal behaviors or psychomotor agitation, a mood-congruent affect, and a goal-oriented, but concrete thought process. Labs and physical exam were unremarkable, with the exception of scars and superficial cuts on bilateral wrists. He was voluntarily admitted to an inpatient psychiatric unit.

Within 24 hours of admission, the patient started an ongoing pattern of signing 72-hour notices for discharge with the sentiment that he needed to leave the hospital to go to work, but shortly after rescinding his request, he stated he needed to prioritize his mental health. Per state guidelines, when a patient is a voluntary admission to a psychiatric hospital, they have the right to sign themselves out; once this form is signed, the treatment team has 72 hours to evaluate the patient to determine if they meet criteria for involuntary psychiatric hospitalization, prompting pursuit of involuntary commitment petition, or prepare patient for discharge with appropriate outpatient follow-up and services. The patient may rescind their 72-hour notice if they change their mind on wanting to leave and decide to continue voluntary treatment. Within the first two days of admission, the patient had two events in which he approached the nursing station and endorsed SI with intent and specified his plans to strangle himself, swallow something (a marker cap) to choke on, or open superficial cuts on his arms. He proceeded to wrap his hands around his neck in one instance and then bite his arm in the other, right in front of the nursing station. The patient revealed to the treatment team that he has another personality that makes him do dangerous things. He stated that it is the other personality who continuously tells him to sign 72-hour notices, harm himself, and make suicidal threats. He clarified that his previously mentioned auditory hallucinations of voices are actually just his other personality telling him to do things.

The patient reported an improvement in his depressive symptoms, auditory hallucinations, and suicidal thoughts over the course of his hospitalization on an adjusted psychotropic medication regimen: olanzapine 5 mg nightly, venlafaxine ER 225 mg daily, and prazosin 2 mg nightly for treatment of psychosis, depression, and PTSD. He opened up to the treatment team that his trauma was from previous psychiatric hospitalizations, but would not further elaborate. The patient had no additional significant events. The patient was discharged on hospital day 9 after he refused to rescind a 72-hour notice despite the treatment team’s recommendations. He did not meet involuntary psychiatric hospitalization criteria at this time.

Second hospital presentation

Five days following discharge, the patient was seen outside of the psychiatric hospital, sitting on the curb, cutting his right forearm superficially. He was brought inside for evaluation. While in the waiting room, he wrapped a hospital gown around his neck to strangle himself and attempted to ingest Band-Aids that were placed over his wrist lacerations. He was immediately admitted and brought back to the unit. While on the unit, he attempted to choke himself with his hands and then bit his right forearm where he had previously been cutting. The patient was verbally de-escalated. Later, when he was asked why he returned to the hospital, he said, “I did not feel like me”. He reported that he did not take his medications upon discharge and was feeling depressed, suicidal, and was hearing voices. He further stated that his other personality was doing things to try to kill him, but did not give additional details.

It was at this time that the patient gave the treatment team permission to speak with his mother, with whom he was living and had a close relationship. She provided additional information on his history of Klinefelter syndrome, early intervention with speech and occupational therapy starting at the age of 10 months until three years, and continued specialist care at an academic medical center with endocrinology, urology, and behavioral health. She reported a history of neuropsychiatric testing and subsequent ADHD and ASD diagnoses as a teenager. She also described his history of hearing voices that previous psychiatrists deemed not psychotic in nature; he had a history of talking to himself and imaginary friends earlier in adolescence. His mother confirmed previous failed psychiatric medication trials but highlighted a previous positive response to propranolol for anxiety that was discontinued for low blood pressure. She talked at length about his social history as he graduated high school with an individualized educational program (IEP), was working at a grocery store for several months, had no drug or alcohol use, and had a positive relationship with his parents and sister. She denied a history of dangerous or self-injurious behaviors at home or at school until recently. She did not believe he had many friends growing up, and none currently. 

The patient agreed to decreasing and then discontinuing olanzapine as there was less concern for psychosis. With stable vital signs and blood pressure throughout previous hospitalization and at the time of intervention, he was started on propranolol 10 mg three times a day for off-label treatment of his anxiety. He reported significant improvements in his mood, anxiety, and suicidal thoughts over the next few days. Contrary to this, he repeated his pattern of signing 72-hour notices and then rescinding them shortly after. On hospital day 17, the patient did not rescind his 72-hour notice and was discharged from the hospital as the treatment team did not believe he met the criteria for involuntary commitment. His psychotropic medication regimen was venlafaxine ER 225 mg daily, prazosin 2 mg nightly, and propranolol 10 mg three times a day. Appropriate outpatient psychiatrist and therapist appointments were scheduled, and his mother agreed to his returning home. She reported an improvement in his mood and anxiety from her perspective during visitation and phone calls.

Third hospital presentation

Seven months later, the patient presented to the emergency department (ED) with psychiatric concerns. He reported SI with the same plans he had mentioned in past hospitalizations. He denied acting on any of these ideas and repeatedly denied intent. He reported that he stopped taking his outpatient prescribed medications (venlafaxine ER 225 mg daily, prazosin 2 mg nightly, and propranolol 10 mg three times a day) and was engaging in self-injurious behavior via superficial cuts on his wrists every few weeks. He stated that he wanted to be admitted to the psychiatric hospital for his safety and medication adjustments. The evaluating psychiatrist encouraged the patient to seek treatment at partial hospitalization or intensive outpatient, as he believed inpatient psychiatric hospitalization was not the appropriate level of care. The patient was discharged from the ED.

Within 24 hours, the patient presented to the ED again with active threats that he would kill himself by cutting his wrists and bleeding out. He continued to uphold his desire for inpatient psychiatric hospitalization for his safety and medication adjustment. After speaking with the patient, the patient’s mother, and the patient’s outpatient psychiatrist, the evaluating psychiatrist decided to pursue voluntary inpatient psychiatric hospitalization. However, once the patient was told that he would not be accepted at the same hospital he was previously admitted to due to a lack of bed availability, he requested to be discharged.

**Figure 1:**
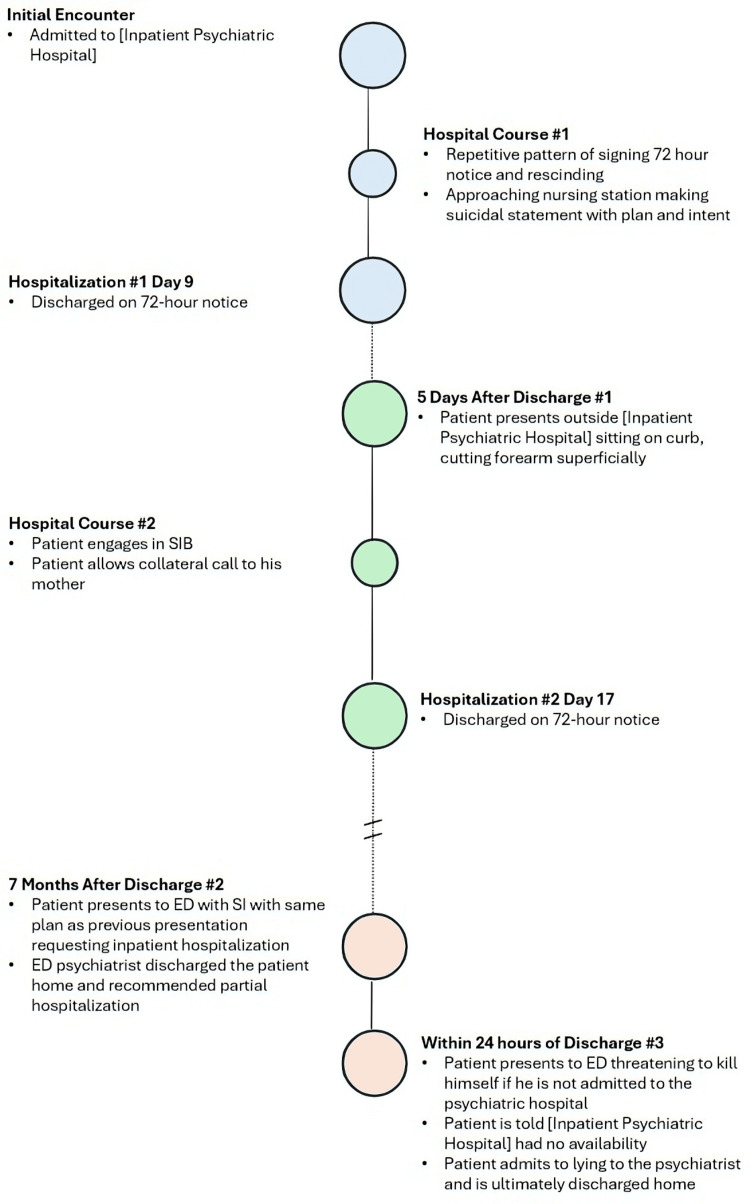
Timeline of case presentation. This case takes place over the course of several months, involving multiple psychiatric presentations. SI: self-ideation; SIB: self-injurious behavior; ED: emergency department.

When asked about his initial reports of SI with plans if he were to be discharged, the patient stated, "I only said that to manipulate the system." He continued by saying, "I knew if I said that, I'd be able to go to (Preferred Inpatient Psychiatric Hospital) because they told me they had a bed available when I called before, but they lied to me." In reference to his self-injurious behaviors, he stated, "I just hurt myself; I actually don't want to end my life." The patient reported that he wanted his medications adjusted, but only at his preferred inpatient psychiatric hospital and at no other facility. At this point, the patient strongly denied current SI with future-oriented thinking, such as goals of continuing classes at the community college and becoming a nurse. He reported feeling safe to be at home going forward and would prefer to go to a partial hospitalization program (PHP) while living with his parents. He was advised to return to the hospital if he were to experience SI and was then discharged with continued psychiatry and psychotherapy with established providers. Figure 1 summarizes the full timeline of this case and its course over several months and multiple psychiatric presentations.

## Discussion

In summary, the patient is a 21-year-old male subject with a history of 48,XXYY syndrome, ASD, and ADHD who presented for psychiatric care with reported SI with several specific plans but without intent; determining the underlying reasoning and potential intent of his ideations challenged the treatment team. This case report presents a complex clinical picture with behaviors that can be associated with ASD, ADHD, and borderline personality disorder (BPD). Specifically, this patient exhibits SI with intent and specific plans, as well as self-injurious behavior. He also demonstrated emotional dysregulation, impulsivity, and instability in decision making throughout the period highlighted by this case, which are core features of BPD, but also seen in ASD and ADHD [8-11].

There is discourse on differentiating the underlying motive for self-injurious behavior in these populations. These include affect-regulation, anti-dissociation, self-punishment, interpersonal influence, anti-suicide, interpersonal boundaries, and sensation-seeking [12]. In BPD specifically, it is thought that these behaviors are utilized to increase the individual’s agency and to cope with negative emotions, such as shame and dissociation, that may result from trauma and social rejection [13-15]. In ASD, difficult behaviors are thought to come from a place of a communication impairment and are seemingly more goal-oriented [16]. Lauren Moskowitz, in collaboration with the Autism Research Institution, discusses in a webinar that individuals with ASD engage in challenging behaviors to communicate a specific need [16]. This may be to request an object, person, activity, or to ask for attention, help, or information [16]. The behaviors may also be to engage in sensory stimulation to lessen feelings of anxiety or to avoid overstimulation and escape certain interactions or demands [16]. This case’s patient had challenging behaviors that seemed to be primarily goal-oriented in nature, such as wanting to be admitted to the psychiatric hospital. Nevertheless, he also had instances of biting and cutting himself outside of this context; this was perhaps to cope with negative emotions or maybe to communicate a different need that was not identifiable. The patient’s report of “another personality that makes him do dangerous things,” but then later clarifying that this other personality talks to him and reporting this as auditory hallucinations, is indicative of probable dissociation rather than psychosis. While not a typical feature of ASD, individuals with ASD can experience co-occurring dissociative symptoms or have difficulty articulating internal experiences in conventional ways [10]. Demonstrating another point of overlap, BPD DSM-5 criteria indicate "transient, stress-related paranoid ideation or severe dissociative symptoms" [17].

The patient’s presentation and subsequent strong response to propranolol also suggest an additional underlying anxious process. There have been several reports demonstrating propranolol as a potential pharmacological agent in managing emotional, behavioral, and autonomic dysregulation for patients with ASD [18]. This case report supports this literature as illustrating another patient with ASD reporting benefit from propranolol. Where this case report deviates from existing literature is the utility of propranolol in individuals with Klinefelter syndrome for anxiety and emotional dysregulation. There are some case reports on propranolol use in Klinefelter syndrome for tremor associated with Parkinsonism, but not for psychiatric treatment [19,20]. Even so, additional studies, specifically randomized clinical trials, would be beneficial to establish standardized propranolol guidelines and dosing.

Aside from a higher ASD prevalence in individuals with 48,XXYY syndrome, the nuances of ASD presentation in this genetic context include distinct cognitive and behavioral profiles, and specific social-communication challenges compared to both the general population, idiopathic ASD, and other sex chromosome aneuploidies. The ASD phenotype is shaped by the underlying neurodevelopmental impact of the extra sex chromosomes, resulting in a profile that is both similar to and distinct from idiopathic ASD and other sex chromosome aneuploidies [7]. An important finding from the study by Tartaglia et al. in 2017, individuals with SCA without an ASD diagnosis had elevated scores on autism screening assessments (Social Communication Questionnaire and Social Responsiveness Scale); in addition, one-third of participants with SCA without ASD met criteria for at least one domain of the Autism Diagnostic Observation Schedule [7]. Overlapping characteristics include language delays, poor eye contact, oversensitivity to sensory stimuli, poor social relatedness, and restricted interests [7].

Similar to ASD, early developmental delays in both motor and language domains are typical, and these delays contribute to the emergence and recognition of ASD features [1]. Motor delays and hypotonia are common, which may delay ambulation [21]. Individuals with 48,XXYY typically have IQs ranging from mild to moderate intellectual disability to the lower end of average, with some adaptive functioning impairment [1]. Visual-spatial skills are often better than verbal abilities, but not necessarily better than those of typically developing peers, with compromised expressive language [20]. Socialization and communication skills are significantly impaired, as measured by standardized tools such as the Vineland Adaptive Behavior Scales [1,22]. This language impairment is a key contributor to ASD-like social communication deficits.

Individuals with 48,XXYY syndrome experience a range of psychosocial and executive functioning challenges, including increased behavioral problems, anxiety, depression, and a higher risk of psychosis in adulthood [7]. Common issues include oppositional defiant disorder, emotional immaturity, obsessive-compulsive behaviors, impulsivity, and tic disorders. Studies highlight tendencies towards confabulation, socialization with younger individuals, and episodic violent acts [7]. Despite these challenges, recent research suggests that psychosocial issues may not be as severe as previously thought, with some positive behavioral features present [7]. Approximately 50% of patients receive psychopharmacological treatment for attention, anxiety, and mood instability, with one-third requiring hospital care. Significant deficits in adaptive functioning were reported [21]. Psychopharmacologic medications and behavioral therapies are effective in managing symptoms [22,23].

MRI studies reveal structural brain differences in 48,XXYY, including reduced frontal and temporal lobe volumes and increased parietal lobe and ventricular volumes [1]. These changes may underlie the observed deficits in executive function, social cognition, and language, all of which may be perceived in ASD presentation. Due to the higher prevalence of ADHD, mood disorders (anxiety, depression), and behavioral dysregulation (hyperactivity, aggression, conduct problems) in individuals with 48,XXYY syndrome, the overlap of these conditions can complicate the clinical picture and may mask or mimic core ASD symptoms [7,24,25].

In summary, individuals with 48,XXYY syndrome have an increased risk for autism symptoms and may meet the criteria for ASD. However, the presentation of autism traits in this population differs from idiopathic autism, where they may not meet criteria for diagnosis and warrant the same services. These distinctions underscore the importance of considering genetic context when understanding the biopsychosocial challenges and psychiatric presentation of individuals with 48,XXYY syndrome, as illustrated in this case report.

## Conclusions

This case report highlights complex psychiatric issues in an individual with 48,XXYY syndrome, ASD, and ADHD, emphasizing the challenges in diagnosis and treatment due to overlapping symptoms and behaviors. The patient's recurrent hospitalizations and varied symptoms, including manipulative behaviors and dissociative experiences, underscore the need for a comprehensive, individualized approach to care. This case illustrates the importance of considering genetic context, particularly the 48,XXYY genotype, in understanding psychiatric manifestations and tailoring treatment strategies. The observed benefits of propranolol in managing anxiety and emotional dysregulation suggest potential avenues for pharmacological intervention and warrant further investigation. Future research should focus on distinguishing the psychiatric profile of 48,XXYY syndrome from other chromosomal variations and exploring effective treatment modalities. Such efforts could enhance clinical understanding and improve outcomes for individuals with this rare genetic condition.
